# Prognostic and Predictive Value of *CCND1*/Cyclin D1 Amplification in Breast Cancer With a Focus on Postmenopausal Patients: A Systematic Review and Meta-Analysis

**DOI:** 10.3389/fendo.2022.895729

**Published:** 2022-06-17

**Authors:** Sarah A. Jeffreys, Therese M. Becker, Sarah Khan, Patsy Soon, Hans Neubauer, Paul de Souza, Branka Powter

**Affiliations:** ^1^ Centre of Circulating Tumour Cell Diagnostics and Research, Ingham Institute of Applied Medical Research, Liverpool, NSW, Australia; ^2^ School of Medicine, Western Sydney University, Campbelltown, NSW, Australia; ^3^ South Western Sydney Clinical School, University of New South Wales, Liverpool Hospital, Liverpool, NSW, Australia; ^4^ Department of Medical Oncology, Bankstown Cancer Centre, Bankstown, NSW, Australia; ^5^ Department of Surgery, Bankstown Hospital, Bankstown, NSW, Australia; ^6^ Department of Obstetrics and Gynaecology, University Hospital and Medical Faculty of the Heinrich-Heine University Düsseldorf, Düsseldorf, Germany

**Keywords:** breast cancer, CCND1, cyclin D1, biomarker, meta-analysis, systematic review, amplification

## Abstract

**Background:**

Up to 80% of breast cancers (BCa) are estrogen receptor positive and current treatments target the estrogen receptor (endocrine therapies) and/or CDK4/6 (CDK4/6 inhibitors). *CCND1* encodes the protein cyclin D1, responsible for regulation of G1 to S phase transition in the cell cycle. *CCND1* amplification is common in BCa and contributes to increased cyclin D1 expression. As there are signalling interactions between cyclin D1 and the estrogen receptor, understanding the impact of *CCND1* amplification on estrogen receptor positive patients’ disease outcomes, is vital. This review aims to evaluate *CCND1* amplification as a prognostic and predictive biomarker in BCa.

**Materials and Methods:**

Publications were retrieved from the databases: PubMed, MEDLINE, Embase and Cochrane library. Exclusion criteria were duplication, publication type, non-English language, *in vitro* and animal studies, not BCa, male BCa, premenopausal BCa, cohort size <35, *CCND1* amplification not reported. Publications with cohort duplication, and inadequate recurrence free survival (RFS) and overall survival (OS) data, were also excluded. Included publications were assessed for Risk of Bias (RoB) using the Quality In Prognosis Studies tool. Statistical analyses (Inverse Variance and Mantel-Haenszel) were performed in Review Manager. The PROSPERO registration number is [CRD42020208179].

**Results:**

*CCND1* amplification was significantly associated with positive estrogen receptor status (OR:1.70, 95% CI:1.19-2.43, p = 0.004) and cyclin D1 overexpression (OR: 5.64, 95% CI: 2.32-13.74, p=0.0001). *CCND1* amplification was significantly associated with shorter RFS (OR: 1.64, 95% CI: 1.13-2.38, p = 0.009), and OS (OR: 1.51, 95% CI: 1.19-1.92, p = 0.0008) after removal of studies with a high RoB. In endocrine therapy treated patients specifically, *CCND1* amplification predicted shorter RFS (HR: 2.59, 95% CI: 1.96-3.41, p < 0.00001) and OS (HR: 1.59, 95% CI: 1.00-2.49, p = 0.05) also after removal of studies with a high RoB.

**Conclusion:**

While a lack of standardised approach for the detection of *CCND1* amplification is to be considered as a limitation, *CCND1* amplification was found to be prognostic of shorter RFS and OS in BCa. *CCND1* amplification is also predictive of reduced RFS and OS in endocrine therapy treated patients specifically. With standardised methods and cut offs for the detection of *CCND1* amplification, *CCND1* amplification would have potential as a predictive biomarker in breast cancer patients.

**Systematic Review Registration:**

https://www.crd.york.ac.uk/prospero/, identifier CRD42020208179.

## Introduction

**Figure 1:**
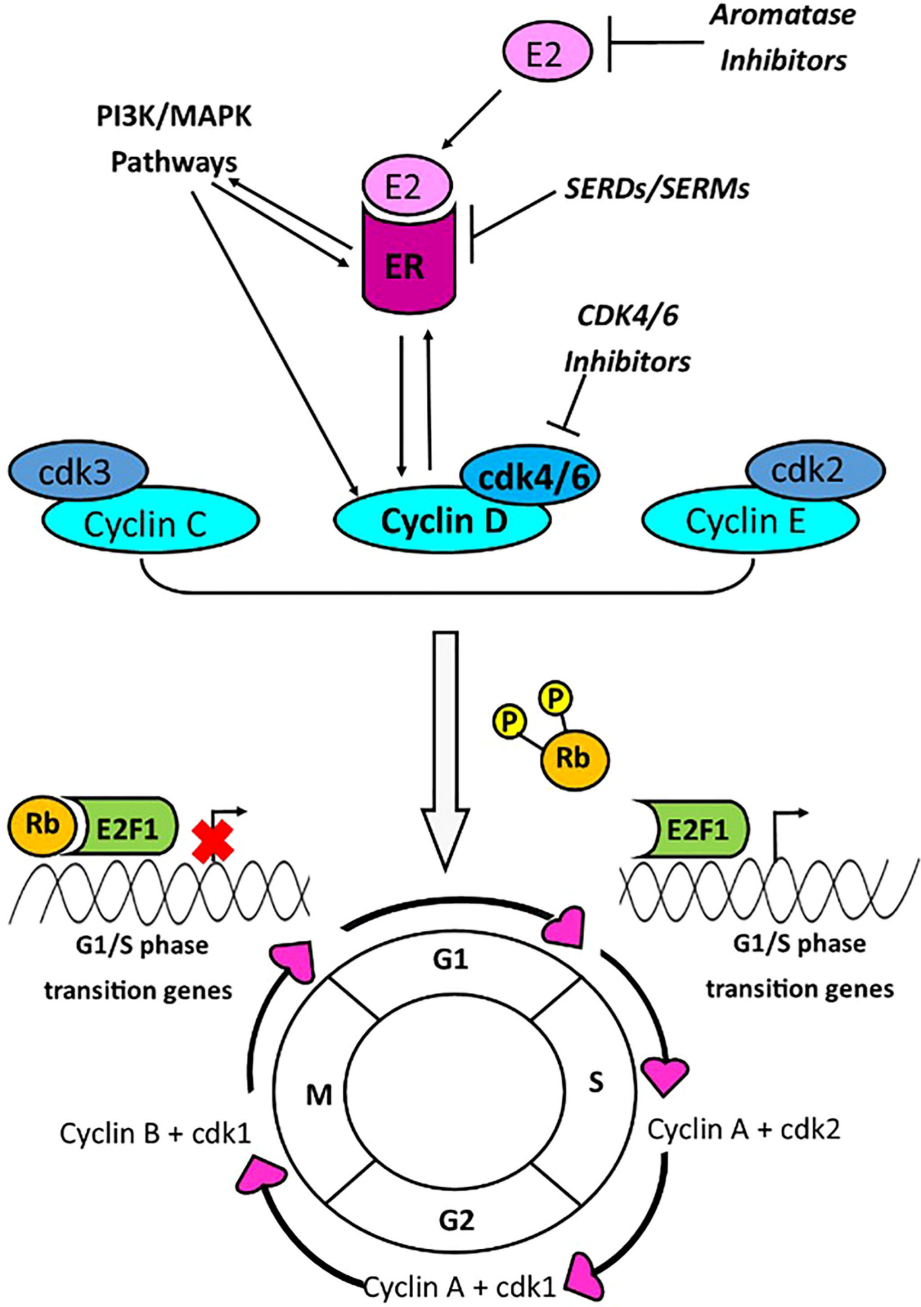
Cyclin D1 promotes G1/S phase cell cycle progression *via* interaction with CDK4/6. Transition between phases of the cell cycle is mediated by cyclins A-E, and cyclin dependent kinases (cdk) 1-6. Cyclins C-E are responsible for the transition from G1 to S phase of the cell cycle. The cyclin D and cdk4/6 complex, activated by PI3K/MAPK pathways or the estrogen receptor (ER), is a key mediator of G1-S phase transition, and this occurs through phosphorylation of retinoblastoma protein (Rb). Phosphorylation of Rb, results in its dissociation with E2F1, enabling transcription of G_1_/S phase genes. Transcriptional activity of the ER is stimulated by cyclin D1, and the ER may activate the *CCND1* promoter. Current breast cancer drugs target estrogen production (aromatase inhibitors); the estrogen receptor [Selective Estrogen Receptor Modulators (SERMs) or Selective Estrogen Receptor Degraders (SERDs)]; or cdk4/6 (cdk4/6 inhibitors).

Chronic sustained cell proliferation is one of the hallmarks of cancer, which is achieved through signalling changes resulting in progression through the cell cycle (1). Cyclin D1, along with its binding partners cyclin dependent kinases (CDK4/6), is a key regulator of the cell cycle, mediating transition from G_1_ to S phase (**Figure 1**). The gene encoding cyclin D1, *CCND1*, located on chromosome 11q13.3, has been reported to be amplified in 10-35% of breast cancers (BCa) (2–4) and its amplification has been associated with increased cyclin D1 expression (3). *CCND1* amplification may be an effective prognostic and predictive biomarker in BCa.

Progression through the cell cycle is regulated by several cyclins and cyclin dependent kinases, at each stage of cycle. During the G_0_ phase, retinoblastoma protein (Rb) inhibits the E2F transcription factor 1 (E2F1), thereby preventing transcription of G_1_/S phase genes (5). The Cyclin D1-CDK4 or 6 complex phosphorylates the Rb, leading to disassociation of Rb from E2F1, thus activating G_1_/S phase gene transcription and cell cycle progression (5) (**Figure 1**). Growth factor signalling pathways, PI3K-AKT-mTOR and MAPK, are linked with both cyclin D1 and Estrogen Receptor (ER) activity (6, 7). There is interplay between the ER, *CCND1* gene and cyclin D1 protein whereby the ER promotes transcription of the *CCND1* gene, and the cyclin D1 protein interacts with the ER to promote ER mediated transcription (8–10).

The ER is expressed in approximately 75% of all BCa tumours (11). *CCND1* amplification is particularly common in ER positive tumours and is associated with reduced survival in these patients (3, 12–15). Many ER positive patients are treated with endocrine therapies (ET), that inhibit the ER pathway, however up to 50% of patients develop resistance (16, 17). *CCND1* amplification is a proposed mechanism of ET resistance, however this remains controversial due to studies with conflicting results. Some studies have shown significant association between *CCND1* amplification and poor aromatase inhibitor (AI) (18, 19) and tamoxifen response (19), whilst another study has shown no association with tamoxifen response (20). Yet, another study suggested *CCND1* was predictive of resistance to aromatase inhibitors but not to tamoxifen (21).

Added to the complexity of these biological relationships, is the role of cyclin D1 overexpression. Whilst the *CCND1* gene amplification is detected in 10-35% of patients (2–4), 50-70% overexpress the cyclin D1 protein, suggesting additional mechanisms of cyclin D1 regulation (14). In a study that separated patients according to *CCND1* amplification status and cyclin D1 overexpression; they found that patients with *CCND1* amplification had reduced Recurrence Free Survival (RFS) compared to those with normal *CCDN1*, but patients with cyclin D1 overexpression had longer RFS than those without (22). Some studies have reported correlation between cyclin D1 expression and poor prognosis in ER positive BCa (23–25) but not all (26, 27). A meta-analysis concluded that cyclin D1 overexpression was a significant predictor of poor prognosis, in ER positive BCa but this was not observed in unselected BCa patients (25). The effects of cyclin D1 expression may differ due to differences in active signalling mechanisms between patients (26, 28).

The role of *CCND1* amplification in BCa, in relation to ET response, RFS and overall survival (OS) remains unclear. This systematic review and meta-analysis evaluates the prognostic and predictive value of *CCDN1* amplification in BCa patients across studies.

## Methods

### Protocol and Registration

This review was registered with PROSPERO: International Prospective Register of Systematic Reviews, registration number [CRD42020208179].

### Information Sources and Search

Publications for screening were obtained from PubMed, MEDLINE, Embase, and Cochrane library databases. These databases were searched on 31^st^ of August 2020 with the search: (“breast cancer” OR “breast carcinoma” OR “breast tumour” OR “breast tumor” OR “breast neoplas*” OR “mammary cancer” OR “mammary carcinoma”) AND (“Cyclin D1” OR CCND1 OR PRAD1 OR BCL1 OR U21B31 OR D11S287E) AND (“Hormone receptor” OR “estrogen receptor” OR “oestrogen receptor” OR ER OR “progesterone receptor” OR PR) AND (Amplification OR “copy number”). There were no restrictions on year of publication. The results from these searches were uploaded to the Rayyan Qatar Computing Research Institute (QCRI) systematic review application (29).

### Study Eligibility and Study Selection

Publications were screened within the Rayyan QCRI (29) platform, by two blinded investigators. Publication duplicates, non-English language publications, reviews, comments, conference abstracts and letters were excluded. In one case, the publication could not be accessed and was excluded under publication type. Other exclusions were studies reporting only *in vitro* or animal findings, not breast cancer, less than 35 participants, premenopausal patients only, male participants only, those that did not report *CCND1* amplification findings. Studies focused on premenopausal and male BCa patients were thus excluded to reduce intra study heterogeneity resulting from biological and treatment differences. For example, premenopausal patients typically present at later stages, have worse long-term outcomes, and receive different therapeutic regimes (particularly in terms of aromatase inhibitor treatment) than postmenopausal patients (30, 31). Following exclusion, investigators were unblinded, and any discrepancies were resolved by consensus. There were 83 publications remaining, and these were assessed first for cohort duplication. Studies that were deemed as having cohorts of the same patients were grouped and the study with the most patients was selected for inclusion; where they had the same number of patients, the most recent study was selected. There were two studies which each had two cohorts of patients, and one of these cohorts was the same in both studies; in this case, data was extracted from both cohorts of one study (32) and only from the non-duplicated cohort from the second study (14). Data extraction was performed on the remaining 69 studies, and these studies were then screened for availability of survival data for analysis of hazard ratios (HR). A final number of 18 studies were included, since the remainder did not provide sufficient survival data for analysis. Of these 18 studies, only those providing the necessary data for each analysis were included, and therefore the total number of studies included in each analysis differs, as reported in the results section.

### Data Collection

Publications were uploaded to Covidence (33), which enabled data extraction using a customisable data extraction form, by one reviewer. Collected data included: general information (title, study type, cohort size, recruitment dates and place, cohort size, inclusion and exclusion criteria), patient characteristics (type of breast cancer, ER status, menopausal status, treatment type), *CCND1* detection (method of detection and cut off), cyclin D1 expression (method, cut-off, correlation with *CCND1* amplification) and *CCND1* amplification correlation with other factors (human epidermal growth factor receptor 2 (HER2), ER, and progesterone receptor (PR) status, histological grade, clinical stage and treatment) and finally, *CCND1* and outcomes: OS, breast cancer specific survival (BCSS), disease free survival (DFS) and recurrence free survival, relapse free survival, time to progression, and recurrence event numbers.

### Risk of Bias

A Risk of Bias (RoB) assessment was performed on all 18 studies included in the meta-analysis, by two blinded investigators, using a customisable quality assessment form in Covidence (33). The quality assessment form was customised to align with the Quality in Prognosis Studies (QUIPs) tool (34). This tool assesses bias across six domains, namely: study participation, study attrition, prognostic factor measurement, outcome measurement, study confounding, and statistical analysis and reporting (34). Study participation was assessed with focus on inclusion and exclusion criteria, number of patients in cohort, and reported characteristics of the cohort. Study attrition focussed on the proportion of samples not assessed for *CCND1* amplification, and if reasons were provided for sample loss. For prognostic factor measurement, whether appropriate methods and controls were used for detection of *CCND1* amplification, and if cut-offs were reported, were considered. Outcome measurement was assessed based on definitions of survival data reported, whether these were considered standard, and reporting of follow up times. Study confounding considered other clinicopathological features, statistical comparisons made between these and *CCND1* amplification, and how stage and grade were assessed. For assessment of statistical analysis and reporting, reporting and type of statistical test were considered, as well as the proportion of *CCND1* amplification and survival data reported. Each domain was rated as “low”, “moderate” or “high” RoB, and where insufficient information was provided for judgement, these were rated as “unclear”. Discrepancies between ratings of the investigators were resolved by consensus. Each study was then given an overall rating based on a method reported by Jermy et al, whereby low bias studies have ≤2 domains rated as moderate with the remainder rated low; moderate bias studies have either 3 moderate ratings or one moderate and one high with the remainder rated low; and high bias studies having either ≥2 moderate plus one high rating, or ≥2 high ratings, or ≥4 of moderate ratings for each domain (35).

### Statistical Analysis

Statistical analyses were performed using Review Manager (36). For statistical analyses, studies rated as low and moderate overall RoB are grouped together, whilst studies with high RoB are grouped separately, as indicated in the relevant results sections. The Mantel-Haenszel method was used for statistical analysis of *CCND1* amplification and clinicopathological features (ER, PR, HER2, stage, grade, and cyclin D1 expression). Statistics for *CCND1* amplification and clinicopathological features are expressed as OR as these are categorical variables. Some analyses required grouping of data for analysis. For example, for grade, grades I-II were combined and compared with grade III; for stage T1-T2 were combined and compared with T3-T4, and for cyclin D1 overexpression low was compared to moderate and high combined; these categories were as reported by individual studies. The definitions used for grade and stage were considered as part of the RoB outcome measurement assessment. For the analysis of OS and RFS, the inverse-variance method was used. In these analyses, statistics were reported as HR as these are continuous variables. A fixed effects approach was taken for analyses with I^2^ <50%, whilst a random effect approach was used when I^2^ was >50%. OS analysis included both OS and BCSS. RFS analysis included recurrence free survival, relapse free survival, disease free survival and recurrence events raw data. Where HR and standard error (SE) were not provided, these were calculated based on provided summary statistics, in accordance with previously described methods (37). In each analysis, all studies reporting the necessary data for that specific analysis were included.

## Results

### Results of Search and Included Studies

**Figure 2:**
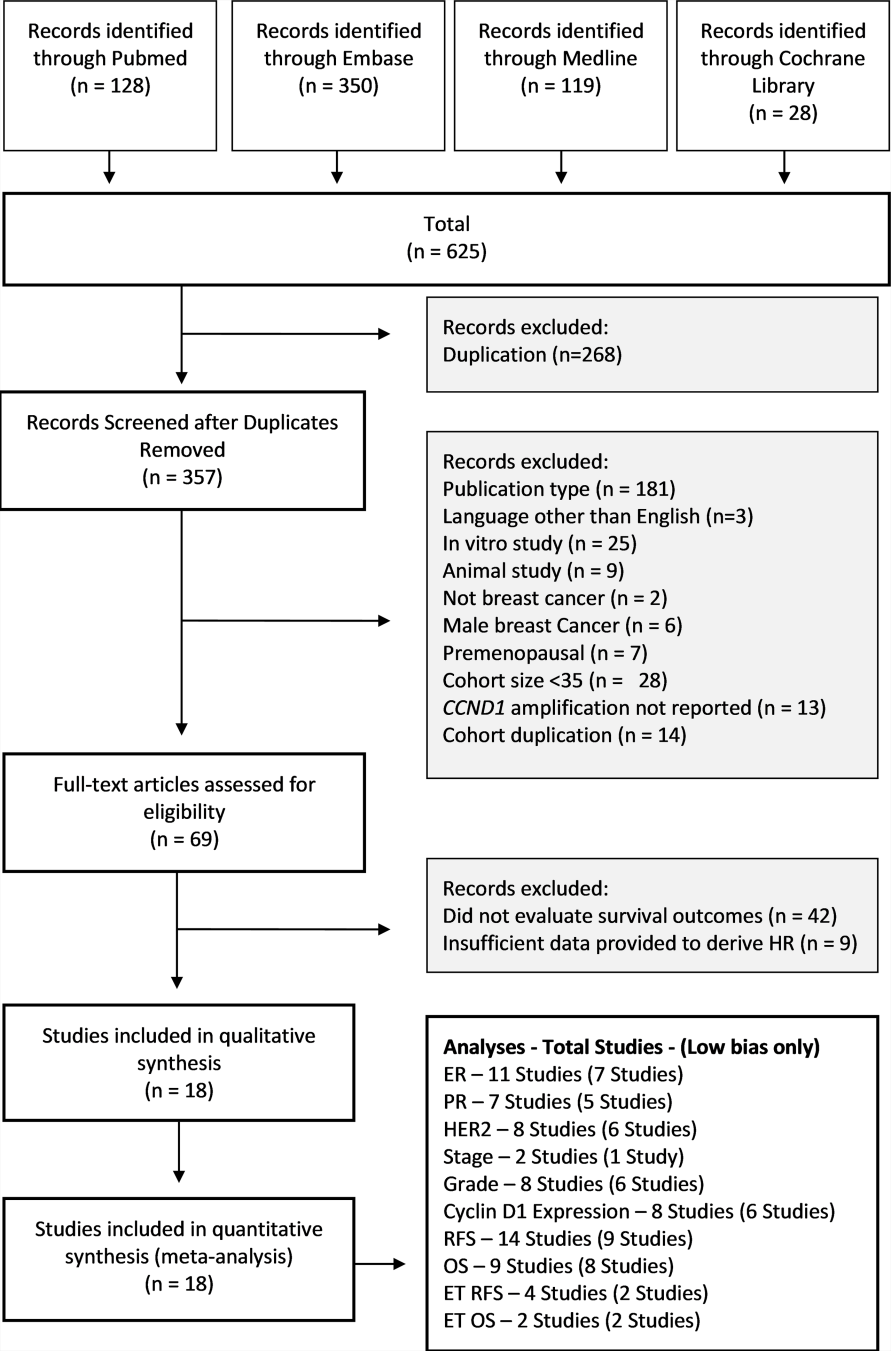
Study Selection PRISMA Diagram. Flow diagram shows studies retrieved from databases, and the number of studies excluded and the basis on which they were excluded. OS, Overall Survival; RFS, Relapse Free Survival; HR, Hazard Ratio.

The process of exclusion and inclusion of studies is summarised in **Figure 2**. Across the four databases, 625 results were retrieved, of which 268 were duplicates. During screening, studies were excluded for the following reasons: publication type (181 studies), language other than English (3 studies), *in vitro* study (25 studies), animal study (9 studies), male breast cancer (6 studies), premenopausal (7 studies), cohort size <35 patients (28 studies), *CCND1* amplification not reported (13 studies) and cohort duplication (14 studies). After screening the remaining studies, 69 studies were deemed eligible for further evaluation. Of these 69 studies, 42 were excluded as they did not evaluate RFS or OS, and nine were excluded due to insufficient summary data to derive HR. The remaining 18 studies formed the basis for this meta-analysis.

### Risk of Bias Assessment

Results of quality assessment, using the QUIPs tool, are shown in **Table 1**. There were three studies (22, 42, 49) that were deemed to have a low RoB across all six domains. All 18 studies were rated as low RoB for study participation, with all of them describing inclusion or exclusion criteria and all reporting key study characteristics. For study attrition, four studies (3, 19, 41, 45) had >20% of the cohort samples not assessed for *CCND1* amplification, and four (26, 38, 43, 45) did not provide adequate reasoning for sample loss. For prognostic factor measurement, two studies (19, 44) inadequately reported methods and controls used for *CCND1* amplification detection assays, however, all studies reported *CCND1* amplification copy number cut-offs. For outcome measurement, definitions of OS and RFS were considered as well as whether follow up time was reported. Definitions of OS or RFS were not reported in six studies (20, 41, 44, 46–48), as such, for these six studies, plus an additional study (45), it was unclear whether the method of RFS and OS measurement were standard (outcome measurement). Also, for outcome measurement, four studies (20, 32, 46, 48) failed to report follow up time. For the study confounding domain, two studies (39, 46) did not adequately measure important confounders, six studies (19, 20, 32, 39, 45, 46) did not define grade or stage measurements, and five (39, 41, 43, 45, 46) made no statistical comparisons between grade and stage and either prognostic or outcome measurements. For the statistical analysis and reporting domain, two studies did not perform statistical analysis of raw data for *CCND1* amplification and RFS or OS (39, 46), six studies did not adequately report statistical methods (14, 19, 20, 39, 44, 46) and three studies (20, 39, 46) selectively reported *CCND1* amplification and survival data. There were four studies that were assessed as having a high overall RoB (19, 20, 45, 46); in each analysis these are represented in a separate subgroup.

**Table 1:** Quality Assessment of Eligible Studies.

Study Ref	Study Participation	Study Attrition	Prognostic Factor Measurement	Outcome Measurement	Study Confounding	Statistical Analysis and Reporting	Overall Rating
**Beelen 2018** (38)	Low	Moderate	Low	Low	Low	Low	Low
**Bièche 2002** (22)	Low	Low	Low	Low	Low	Low	Low
**Bostner 2007** (20)	Low	Low	Low	High	Moderate	Moderate	High
**Cao 2019** (39)	Low	Low	Low	Low	High	Moderate	Moderate
**Carene 2020** (32)	Low	Low	Low	Moderate	Moderate	Low	Low
**Elsheikh 2008** (3)	Low	Moderate	Low	Low	Low	Low	Low
**Hadzisejdic 2010** (40)	Low	Low	Low	Low	Low	Low	Low
**Kirkegaard 2008** (41)	Low	Moderate	Low	Moderate	Moderate	Low	Moderate
**Lundberg 2019** (14)	Low	Low	Low	Low	Low	Low	Low
**Lundgren 2012** (19)	Low	Moderate	Moderate	Low	Moderate	Moderate	High
**Massidda 2010** (42)	Low	Low	Low	Low	Low	Low	Low
**Muss 2007** (43)	Low	Moderate	Low	Low	Low	Low	Low
**Ortiz 2017** (26)	Low	Moderate	Low	Low	Low	Low	Low
**Plevova 2010** (44)	Low	Low	Moderate	Moderate	Low	Low	Low
**Quintayo 2012** (45)	Low	High	Low	Low	High	Low	High
**Serino 2019** (46)	Low	Low	Low	High	High	High	High
**Seshadri 1996** (47)	Low	Low	Low	Moderate	Low	Low	Low
**Tabarestani 2014** (48)	Low	Unclear	Low	High	Low	Low	Moderate

Low, moderate, and high refer to the level of risk of bias for each domain, for each study.

Overall Rating Scoring System (35): Low risk: Up to 2 moderate ratings with remainder low. Moderate risk: 3 low + 3 moderate OR 1 moderate + 1 high. High risk: ≥2 moderate + 1 high OR ≥2 high, OR ≥4 moderate.

### Characteristics of Included Studies

Characteristics of studies are summarised in **Table 2**. *CCND1* amplification was detected by various methods including: Florescence *In Situ* Hybridisation (FISH) (26, 41–45, 49, 50), Chromogenic *In Situ* Hybridisation (CISH) (3, 19), RT-PCR (20, 22, 46), Multiplex Ligation-dependent Probe Amplification (MLPA) (14, 38), Targeted Sequencing (32), Nanostring copy number variation assay (39), Southern Blot (22), Slot Blot Hybridisation (47) (**Table 2**). Amplification cut-offs varied significantly between studies, even amongst those with the same detection method. Some studies counted number of copies, some a copy number ratio relative to a control gene, some considered the number of signals and the proportion of cells with signals, whilst others had unique measures based on their method and corresponding controls. The frequency of *CCND1* amplification ranged from 9%-57%. The studies collectively comprised 6400 patient samples and of these, 1135 (18%) were considered *CCND1* amplified.

**Table 2:** Characteristics of Eligible Studies.

Study Ref	Method	*CCND1* Amplification Cutoff	*CCND1* Amplification CutoffDetails	*CCND1* Amplified (n= 1135)	Cohort Size (n= 6400)	Outcomes Measured
**Beelen 2018** (38)	Multiple ligation-dependent probe amplification	> 0	Log 2 copy number ratio	271 (57%)	476	OS
**Bièche 2002** (22)	94 samples RT-PCR All samples Southern Blot	>2.5 >2.0	Fold difference from reference and calibrator	15 (15%)	102	RFS
**Bostner 2007** (20)	RT-PCR	>3.6	Copy number ratio	28 (12%)	226	RFS
**Cao 2019** (39)	Nanostring copy number variation assay	≥5	Copy number calls	23 (33%)	70	RFS
**Carene 2020** (32)	Targeted Sequencing	>4	Copies	70 (21%)	327	DDFS
**Elsheikh 2008** (3)	CISH	>5	Signals per nucleus in more than 50% of cancer cells, or when large gene copy clusters were seen.	49 (10%)	475	OS
**Hadzisejdic 2010** (40)	FISH	≥2	Copy number ratio	15 (13%)	112	OS
**Kirkegaard 2008** (41)	FISH	≥2	Copy number ratio	73 (21%)	354	OS
**Lundberg 2019** (14)	Multiplex ligation-dependent probe amplification	>3.0	Genomic Identification of Significant Targets in Cancer G-Score	119 (35%)	340	BCSS
**Lundgren 2012** (19)	CISH	≥ 1	Copies	101 (9%)	1155	TTR
**Massidda 2010** (42)	FISH	≥ 3	Signals in at least 10% of nuclei.	12 (23%)	53	DFS, OS
**Muss 2007** (43)	FISH	≥2	Copy number ratio	16 (14%)	112	RFS
**Ortiz 2017** (26)	FISH	≥6	Copies in >50% of the cells	34 (19%)	179	DFS, OS
**Plevova 2010** (44)	FISH	≥1.5	Copy number ratio	8 (30%)	33	DFS, OS
**Quintayo 2012** (45)	FISH	>2	Copy number ratio	146 (14%)	1076	DRFS
**Serino 2019** (46)	RT-PCR	>2.5	Copy number ratio	14 (30%)	46	Events*
**Seshadri 1996** (47)	Slot Blot Hybridisation	>2	Copy number ratio	103 (9%)	1094	RFS, OS
**Tabarestani 2014** (48)	FISH	>1.3	Peak Values	38 (22%)	170	RFS

PCR, Polymerase Chain Reaction; FISH, Fluorescence In Situ Hybridisation; CISH, Chromogenic In Situ Hybridisation; OS, Overall Survival; RFS, Recurrence Free Survival; DDFS, Distant Disease Free Survival; BCSS, Breast Cancer Specific Survival; TTR, Time to Recurrence, DFS, Disease Free Survival. *Events included: Metastasis, Local relapse, Contralateral Breast Cancer, Death.

### *CCND1* Amplification and Clinicopathological Features

Several studies provided sufficient data for analysis of *CCND1* amplification status and clinicopathological features, including ER (11 studies), PR (7 studies), HER2 status (8 studies), tumour stage (2 studies), histologic grade (8 studies) as well as cyclin D1 expression (8 studies). *CCND1* amplification was significantly associated with ER status (OR: 1.70, 95% CI: 1.19-2.43, p = 0.004) and cyclin D1 overexpression (OR: 5.64, 95% CI: 2.32-13.74, p = 0.0001) (**Table 3** and **Supplementary Figures 1, 2**). There was low heterogeneity (I^2^ = 35%, p = 0.13) for ER analysis, but high (I^2^ = 88%, p < 0.00001) for cyclin D1 overexpression analysis. *CCND1* amplification was not significantly associated with: PR (p = 0.71) or HER2 (p = 0.39) status, tumour stage (p = 0.20) or histologic grade (p = 0.28) (**Table 3** and **Supplementary Figures 3–6**). Analysis of *CCND1* amplification and clinicopathological features excluding studies that had a high RoB did not differ substantially from the analysis of all eligible studies (**Supplementary Figures 1–6**).

**Table 3:** *CCND1* Amplification and Clinicopathological Features.

Clinicopathological Features	Statistical Method	Heterogeneity		OR	95% CI	p-Value
		p-Value	I^2^ (%)			
ER+ vs. ER-	Random Effects, Mantel-Haenszel	0.13	35%	1.70	1.19-2.43	**0.004**
PR+ vs. PR-	Random Effects, Mantel-Haenszel	0.12	41%	1.06	0.78-1.43	0.71
HER2+ vs. HER2-	Random Effects, Mantel-Haenszel	0.24	24%	0.84	0.56-1.26	0.39
Stage T1 & T2 vs. T3 & T4	Random Effects, Mantel-Haenszel	0.71	0%	0.70	0.41-1.21	0.20
Grades I & II vs. Grade III	Random Effects, Mantel-Haenszel	0.06	48%	1.21	0.86-1.70	0.28
Cyclin D1 Negative/Low vs Moderate/High	Random Effects, Mantel-Haenszel	<0.00001	88%	5.64	2.32-13.74	**0.0001**

ER, Estrogen Receptor; PR, Progesterone Receptor; HER2, Human Epidermal Growth Factor Receptor 2; OR, Odds Ratio; CI, Confidence Interval. Corresponding forest plots are provided in supplementary data. Bold values indicate significant p-value.

### *CCND1* Amplification and Recurrence Free Survival

**Figure 3:**
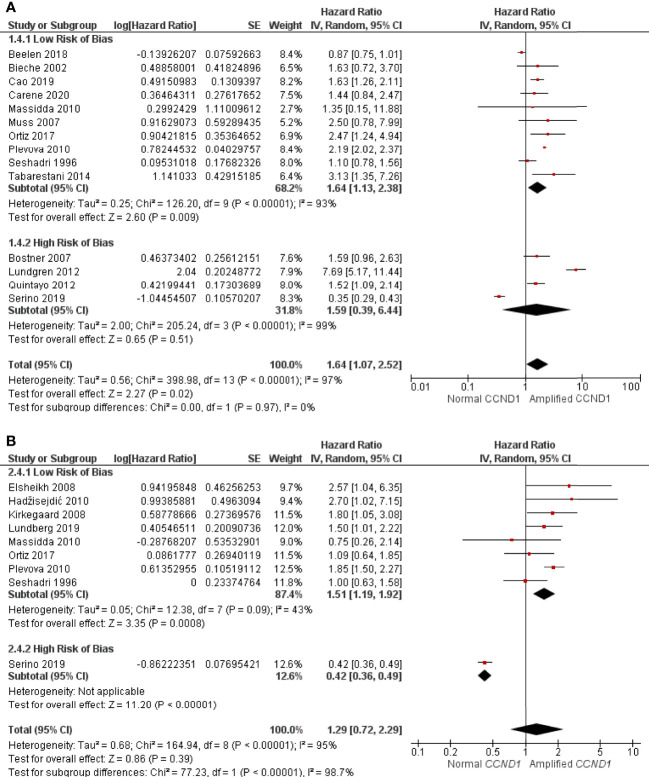
Forrest plot of hazard ratios for *CCND1* amplification and worse relapse free survival and overall survival of breast cancer patients. **(A)** *CCND1* Amplification and relapse free survival **(B)** *CCND1* Amplification and overall survival. Z values indicate the magnitude of association, with p-values <0.05 indicating statistically significant association. Red squares indicate hazard ratio, with values >1 indicative of association of the outcome measure (RFS and OS) with *CCND1* amplification, with strongest association towards the right of the plot. Black lines either side of squares indicate 95% confidence interval (CI). Size of red boxes is relative to specific study weight with greatest weight given to studies with minimal variance (calculated based on inverse of the variance). Large black diamond represents pooled hazard ratio estimate of the above studies. A random effects approach was taken. SE, Standard Error. Plots were generated in Review Manger.

A total of 14 studies consisting of 5083 patients had sufficient data for inclusion in the RFS analysis. *CCND1* amplification was found be associated with significantly worse RFS (HR: 1.64, 95% CI: 1.07-2.52, p = 0.02), with high heterogeneity (I^2^ = 97%, p < 0.00001) (**Figure 3A**). Of the 14 studies, four studies, consisting of 2503 patients, were assessed as having a high RoB; exclusion of these studies did not substantially alter association between *CCND1* amplification and RFS (HR: 1.64, 95% CI: 1.13-2.38, p = 0.008) with high heterogeneity (I^2^ = 93%, p < 0.00001) (**Figure 3A**).

### *CCND1* Amplification and Overall Survival

Nine studies consisting of 2697 patients reported sufficient OS data for statistical analysis. There was no statistically significant association between *CCND1* amplification and worse OS (HR: 1.29, 95% CI: 0.72-2.29, p = 0.39) with the high heterogeneity (I^2^ = 95%, p < 0.00001) (**Figure 3B**). Of these nine studies, one consisting of 56 patients, was considered as having a high RoB. After excluding this study, eight studies of 2641 patients remained for reanalysis. This reanalysis showed a statistically significant association between *CCND1* amplification and worse OS (HR: 1.51, 95% CI: 1.19-1.92, p = 0.0008) with low heterogeneity (I^2^ = 43%, p = 0.09) (**Figure 3B**).

### *CCND1* Amplification and Endocrine Therapy

**Figure 4:**
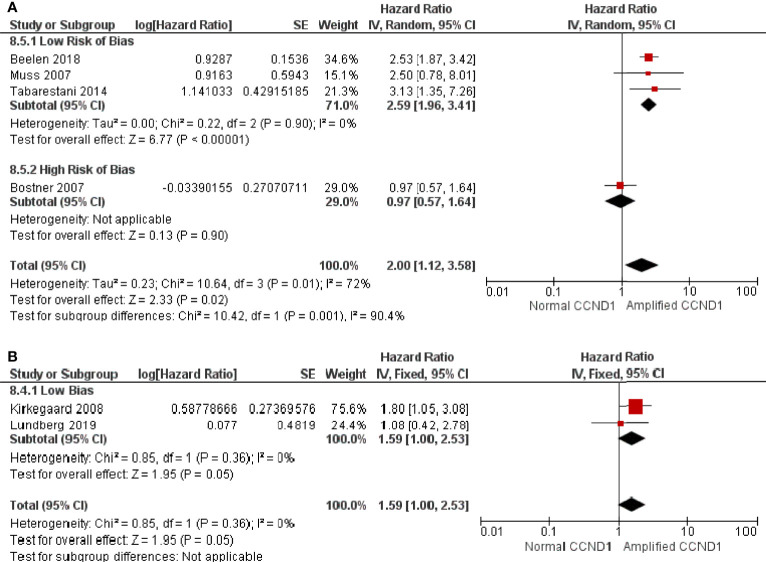
Forrest plot of hazard ratios for *CCND1* amplification and worse relapse free survival and overall survival of endocrine therapy breast cancer patients.**(A)** *CCND1* Amplification and relapse free survival **(B)** *CCND1* Amplification and overall survival. Z values indicate the magnitude of association, with p-values <0.05 indicating statistically significant association. Red squares indicate hazard ratio, with values >1 indicative of association of the outcome measure (RFS and OS) with *CCND1* amplification, with strongest association towards the right of the plot. Black lines either side of squares indicate 95% confidence interval (CI). Size of red boxes is relative to specific study weight with greatest weight given to studies with minimal variance (calculated based on inverse of the variance). Large black diamond represents pooled hazard ratio estimate of the above studies. A random effects approach was taken. SE, Standard Error. Plots were generated in Review Manger.

There were four studies, comprised of 1083 patients, that reported *CCND1* amplification and RFS in patients on endocrine therapy. Patients with *CCND1* amplification had significantly shorter RFS whilst on endocrine therapy than those without amplification (HR: 2.00, 95% CI: 1.12-3.58, p=0.02) with high heterogeneity (I^2 ^= 72%, p=0.01) (**Figure 4A**). One of the studies of 226 patients was deemed as having a high RoB, and their exclusion resulted in a greater effect (HR: 2.59, 95% CI: 1.96-3.41, p<0.00001) with low heterogeneity (I^2 ^= 0%, p = 0.90) (**Figure 4A**). There were two studies, comprised of 694 patients, that reported *CCND1* amplification and OS in patients on endocrine therapy, and these were assessed as having a low RoB. Patients with *CCND1* amplification had significantly shorter OS whilst on endocrine therapy than those without (HR: 1.59, 95% CI: 1.00-2.49, p = 0.05) with low heterogeneity (I^2^ = 0%, p = 0.36) (**Figure 4B**).

## Discussion

*CCND1* is an oncogene that encodes the protein cyclin D1. Cyclin D1, in conjunction with CDK4/6, promotes progression through the cell cycle from G_1_ to S phase. Amplification of *CCND1* is a proposed marker of poor prognosis, in BCa, and in some studies is associated with ET resistance. Despite several studies investigating the predictive and prognostic value of *CCND1* amplification, much ambiguity remains.

This meta-analysis included 18 studies comprising 6400 patients. Of these, 1136 (18%) had *CCND1* amplified tumours, which is within the 10-35% range generally reported (2–4). Previous evidence suggested an association between *CCND1* amplification and ER positive status (4, 13, 15, 51). Our analysis supports these findings, demonstrating a strong relationship between *CCND1* amplification and positive ER status, with low heterogeneity. However, regarding other clinicopathological features, there were no significant relationships between *CCND1* amplification and PR or HER2 status, nor with histological grade or tumour stage. Other studies have yielded conflicting results with respect to *CCND1* amplification and PR status, HER2 status and histological grade (13, 15, 51–54). Many have reported no association between *CCND1* amplification and tumour stage (4, 52–54). A previous meta-analysis found significant association between *CCND1* amplification and ER and PR status as well as histologic grade, but no association with HER2 status or stage (4). Whilst this meta-analysis yielded similar results to one by He et al. (4), the included studies differed considerably; this is in part due to the search terms and exclusion criteria used, as well as several studies being published since their review. Our systematic review and metanalysis targeted studies of hormone receptor postmenopausal patients specifically with cohorts consisting of >35 patients and included analysis of *CCND1* amplification and cyclin D1 overexpression.

There are several factors that may contribute to differences observed between studies, these may include variations in detection methods, cut off definitions for clinicopathological features and of *CCND1* amplification (47), as well as the composition of cohorts in terms of molecular and histological subtypes (54, 55). In this study, analysis of *CCND1* amplification in terms of molecular subtypes was not possible as it was not reported for many of the analysed studies, however others have shown *CCND1* amplification is more common in the luminal subtype and associated with worse breast cancer specific OS compared to other subtypes (14, 56).

Our analysis also demonstrated that *CCND1* amplification is strongly associated with high level of cyclin D1 expression. However, there was high heterogeneity between the studies, which may stem from the different methods of detection and scoring of immunohistochemistry results of cyclin D1 expression, as these were not standardised. Nevertheless, previous studies have noted the association between *CCND1* amplification and high levels of cyclin D1 expression (13, 15, 54). Interestingly, one study showed that cyclin D1 immunostaining with an Allred score of >6.5 (57) was predictive of *CCND1* amplification in ER positive BCa patients, with high sensitivity (94.2%) and specificity (87.8%) (13). This is somewhat surprising, given that *CCND1* amplification occurs in 10-35% of BCa tumours (**Table 1**), and cyclin D1 overexpression occurs in 50-70% of BCa tumours (2–4).

Another study found that *CCND1* amplification and cyclin D1 overexpression correlated in ER positive but not ER negative BCa (58). This may be indicative of a positive feedback loop between cyclin D1 and ER, initiated by *CCND1* amplification; cyclin D1 is known to stimulate ER transcriptional activity, whilst the ER forms a complex with Nuclear Factor Kappa B (NF-κB) and the cofactor, Rac Family Small GTPase 3 (RAC3) to promote *CCND1* transcription (59). It has been found that high levels of Cyclin D1 mRNA was associated with positive ER status (24). Under this model, ER likely promotes expression of cyclin D1, which is further augmented by *CCND1* amplification, accounting for the higher prevalence of cyclin D1 overexpression than amplification (22).

Regardless, *CCND1* amplification retains its value as the preferred prognostic marker over cyclin D1 overexpression, due to several contradictory findings regarding the prognostic value of cyclin D1 mRNA and protein overexpression. There are several possible explanations for these discrepancies, i) differences between molecular subtypes, ii) treatment regime iii) mechanisms underlying cyclin D1 overexpression, iv) methodological differences. Because there is greater evidence for the *CCND1* amplification as a prognostic maker, it has been suggested that studies are needed that consider patients with *CCND1* amplification separately to those with cyclin D1 overexpression in the absence of amplification, in a well-defined cohort (60). Indeed, one study segregated patients into three groups; i) unamplified CCND1, cyclin D1 overexpression, ii) normal *CCND1* and iii) *CCND1* amplified and cyclin D overexpressed; they showed that patients with *CCND1* amplification and cyclin D1 overexpression had worse RFS, whilst those with cyclin D1 overexpression but unamplified had good RFS, compared to those with normal *CCND1* (22). This study demonstrated that there are important differences between these groups, that remain to be fully elucidated. Thus, the current evidence favours *CCND1* amplification as the preferred prognostic marker over cyclin D1 transcript and protein expression. Additionally, our focus on *CCDN1* amplification rather than transcript or protein is also based on practical considerations: with improving molecular technologies *CCDN1* amplification testing could easily be moved into diagnostic settings as economic fast turnaround assay.

*CCDN1* amplification is a proposed mechanism of resistance to ET. To examine this further, we analysed the effect of *CCND1* amplification status on RFS and OS in ET treated patients. In these patients, *CCND1* amplification was significantly associated with shorter RFS and OS. Removal of high RoB studies resulted in a stronger association between *CCND1* amplification and RFS in ET treated patients. One of the limitations for these analyses is the number of studies which reported type of treatment with RFS and OS. The overall analysis for ET and RFS contained four studies, and this was further reduced to just two after exclusion of those assessed as having high RoB. Additionally, majority of studies in the ET RFS analysis reported results for treatment with tamoxifen alone, and hence may be biased towards tamoxifen treatment specifically. Comparison of different types of ET in terms of *CCND1* amplification and RFS and OS, was not possible as majority of studies reported on tamoxifen only, and studies of other ETs were lacking. In fact, just one study (14) reported on ET generally, the others all focused on patients treated with tamoxifen. However, our findings agree with previous studies that reported *CCND1* amplification as predictive of poor prognosis in patients treated with ET, including tamoxifen (15, 61) and AIs (12, 14, 19). However, these are not altogether unanimous; one study found that co-amplification of *CCND1* and *EMSY* predicted tamoxifen resistance (62) whereas another found that *CCND1* amplification was predictive of poor response to AIs but not tamoxifen (21). As mechanisms of ET action differ, it is possible that *CCND1* amplification may contribute to resistance in some treatments but not others (63). However, differences may also be due to biological and chance differences between the cohorts, and further investigation is required to fully elucidate differences between subgroups of ET.

To explore the prognostic potential of *CCND1* amplification, we compared duration of RFS and OS between patients with *CCND1* amplified tumours and those without. In the analysis, including all eligible studies, we found that *CCND1* amplification was significantly associated with shorter RFS but not OS. In both analyses there was a high degree of heterogeneity, and this could potentially be attributed to a range of variables. For example, RoB assessment indicated that multiple studies did not always report RFS and OS definitions or follow up times. Additionally, several studies failed to compare histological grade and tumour stage with *CCND1* amplification. In support of this, removal of studies deemed as having a high RoB, yielded statistically significant association between *CCND1* amplification and both RFS and OS, and reduced heterogeneity particularly in the case of OS.

There are some important limitations of the present study that should be considered. One of these is the variation amongst methods used to define *CCND1* amplification and the cut off values. For example, many studies set cut offs based on a reference probe, with reference probes differing between studies, whilst others may select arbitrarily based on number of signals or the proportion of cells with positive signals. This has the potential to influence results in either direction depending on sensitivity of the assay, and on how cut offs were determined. Due to the wide variety of methods, and the overall number of studies included in this meta-analysis, it was not possible to conduct subgroup analyses between the different methods employed. Secondly, treatment regimens were generally poorly reported, making it difficult to compare amplification status with treatment responses. Thirdly, definitions of survival statistics differed in some studies, or were not reported. Fourthly, some studies only provided HR and 95% CI for subgroups of patients in which there was statistical significance; such underreporting of non-significant results, may have led to a bias in favour of associations between *CCND1* and survival outcomes. Lastly, of the 18 studies included in our analysis, just three were rated as having low RoB across all six domains for quality assessment.

A major challenge with interpretation of data is due to the variation of methods used to detect *CCND1* amplification, and variations in the cut-off values. Amplification detection methods include: southern blot, FISH, CISH, silver *in situ* hybridisation (SISH) PCR based (qPCR, quantitative fluorescence PCR, multiplex ligation-dependent probe amplification and droplet digital PCR), targeted sequencing, array comparative genomic hybridisation, and next generation sequencing (64). FISH was the most common detection method used in the literature, but there are potentially better methods. Increasingly, bright field ISH, CISH and SISH, are becoming the preferred method of amplification detection, owing to their increased resolution, and ability to simultaneously view gene amplification and tissue morphology (65). Currently, in the clinical setting, CISH and SISH are the preferred methods of *HER2* amplification detection, with FISH used only in challenging cases (66). Current methods to define *CCND1* amplification have not been taken up into routine diagnostic settings and development of better assays for rapid, reliable, economic, standardised *CCND1* amplification testing are needed to harness the value of this prognostic and predictive biomarker.

In conclusion, our meta-analysis demonstrated that *CCND1* amplification is significantly associated with positive ER status and cyclin D1 overexpression. *CCND1* amplification was also predictive of both shorter RFS, and OS, in ET treated patients. As a prognostic biomarker, our meta-analysis indicated that *CCND1* amplification may be effective in predicting shorter RFS and OS, after quality assessment. The lack of a standardised method of *CCND1* amplification detection remains a considerable limitation, warranting future investigations aimed at establishing a standardised approach. 

## Data Availability Statement

The raw data supporting the conclusions of this article will be made available by the authors, without undue reservation.

## Author Contributions

Conceptualisation, SJ; literature search and screening, SJ and BP; data collection SJ; risk of bias assessment, SJ and BP; Figures and tables, SJ; statistical analyses, SJ; drafting, SJ, BP, and TB; critical review of work, BP, SK, PS, HN, PdS, and TB. All authors contributed to the article and approved the submitted version.

## Funding

This work was supported by a grant (13/TRC/1-01) from the Cancer Institute NSW through the CONCERT Translational Cancer Research Centre, SJ is a recipient of an Ingham Institute PhD Scholarship, generated by the Liverpool Catholic Club. BP is funded through a Clinical Academic Group Seed Grant from the Sydney Partnership for Health, Education, Research and Enterprise (SPHERE).

## Conflict of Interest

The authors declare that the research was conducted in the absence of any commercial or financial relationships that could be construed as a potential conflict of interest.

## Publisher’s Note

All claims expressed in this article are solely those of the authors and do not necessarily represent those of their affiliated organizations, or those of the publisher, the editors and the reviewers. Any product that may be evaluated in this article, or claim that may be made by its manufacturer, is not guaranteed or endorsed by the publisher.
