# Supplementary material for: Prognostic and Predictive Value of CCND1/Cyclin D1 Amplification in Breast Cancer With a Focus on Postmenopausal Patients: A Systematic Review and Meta-Analysis
Source: Front Endocrinol (Lausanne). 2022 Jun 17;13:895729. doi: 10.3389/fendo.2022.895729 (PMC9249016; doi:10.3389/fendo.2022.895729)
Supplement: Supplementary file 1 [file DataSheet_1.docx]

# Supplementary Data


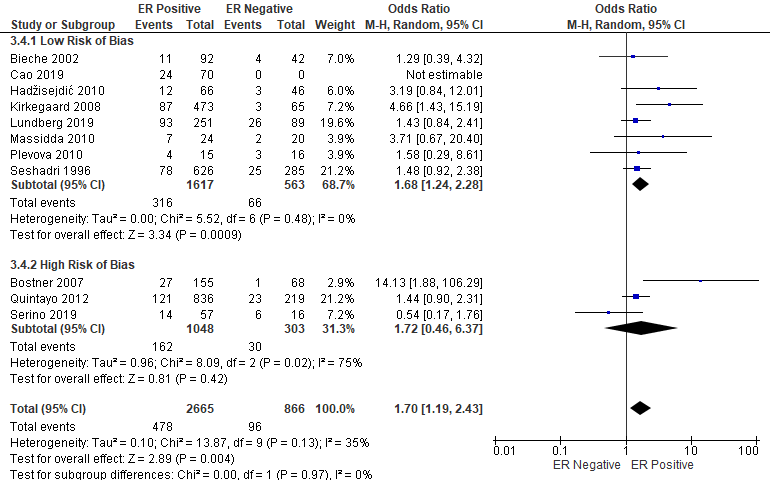


**Supplementary Figure 1: Forrest plot of odds ratios for *CCND1* amplification and Estrogen Receptor**

Analysis includes data from all eligible studies regardless of risk of bias (RoB), with segregation into groups based on RoB assessment. Z values indicate the magnitude of association, with p-values <0.05 indicating statistically significant association. Blue squares indicate ratio, with values >1 indicative of association of the outcome measure (RFS and OS) with *CCND1* amplification, with strongest association towards the right of the plot. Black lines either side of squares indicate 95% confidence interval (CI). Size of blue boxes is relative to specific study weight with greatest weight given to studies with minimal variance (calculated based on inverse of the variance). Large black diamond represents pooled ratio estimate of the above studies. A random effects approach was taken. SE – Standard Error. Plots were generated in Review Manager.

**
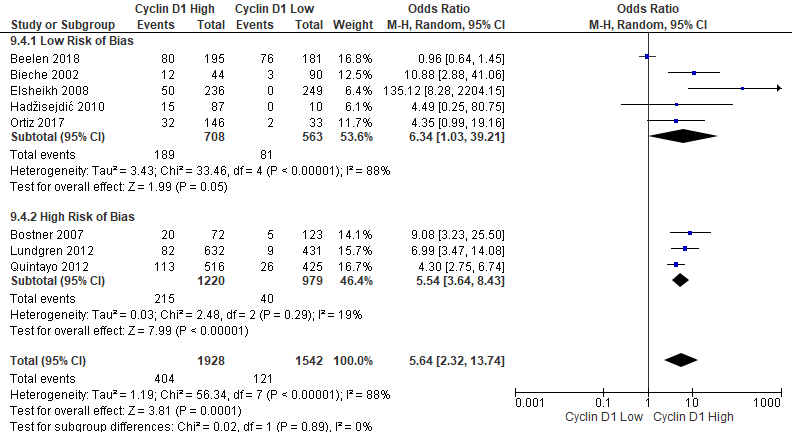
**

**Supplementary Figure 2: Forrest plot of odds ratios for *CCND1* amplification and Cyclin D1 Expression**

Analysis includes data from all eligible studies regardless of risk of bias (RoB), with segregation into groups based on RoB assessment. Z values indicate the magnitude of association, with p-values <0.05 indicating statistically significant association. Blue squares indicate ratio, with values >1 indicative of association of the outcome measure (RFS and OS) with *CCND1* amplification, with strongest association towards the right of the plot. Black lines either side of squares indicate 95% confidence interval (CI). Size of blue boxes is relative to specific study weight with greatest weight given to studies with minimal variance (calculated based on inverse of the variance). Large black diamond represents pooled odds ratio estimate of the above studies. A random effects approach was taken. SE – Standard Error. Plots were generated in Review Manager.

**
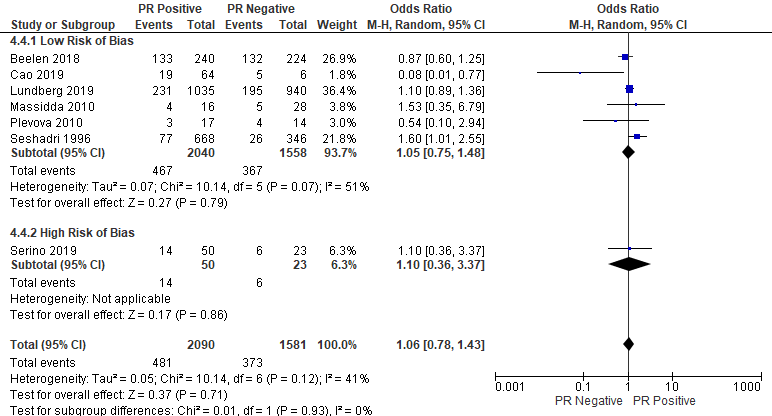
**

**Supplementary Figure 3: Forrest plot of odds ratios for *CCND1* amplification and Progesterone Receptor**

Analysis includes data from all eligible studies regardless of risk of bias (RoB), with segregation into groups based on RoB assessment. Z values indicate the magnitude of association, with p-values <0.05 indicating statistically significant association. Blue squares indicate odds ratio, with values >1 indicative of association of the outcome measure (RFS and OS) with *CCND1* amplification, with strongest association towards the right of the plot. Black lines either side of squares indicate 95% confidence interval (CI). Size of blue boxes is relative to specific study weight with greatest weight given to studies with minimal variance (calculated based on inverse of the variance). Large black diamond represents pooled odds ratio estimate of the above studies. A random effects approach was taken. SE – Standard Error. Plots were generated in Review Manager.

**
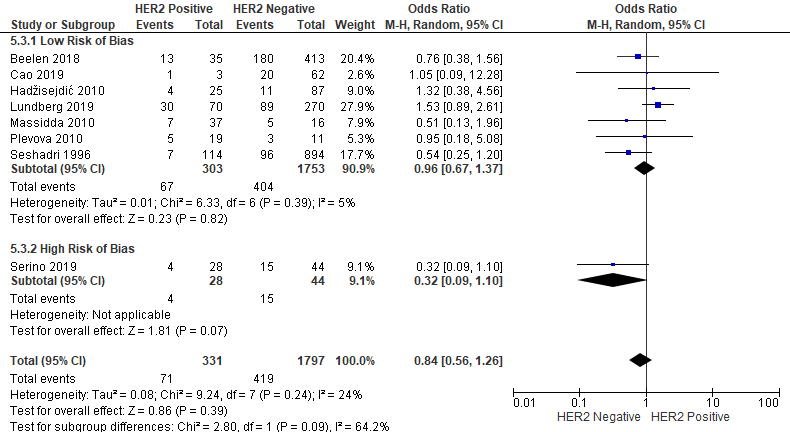
**

**Supplementary Figure 4: Forrest plot of odds ratios for *CCND1* amplification and HER2**

Analysis includes data from all eligible studies regardless of risk of bias (RoB), with segregation into groups based on RoB assessment. Z values indicate the magnitude of association, with p-values <0.05 indicating statistically significant association. Blue squares indicate odds ratio, with values >1 indicative of association of the outcome measure (RFS and OS) with *CCND1* amplification, with strongest association towards the right of the plot. Black lines either side of squares indicate 95% confidence interval (CI). Size of blue boxes is relative to specific study weight with greatest weight given to studies with minimal variance (calculated based on inverse of the variance). Large black diamond represents pooled odds ratio estimate of the above studies. A random effects approach was taken. SE – Standard Error. Plots were generated in Review Manager.

**
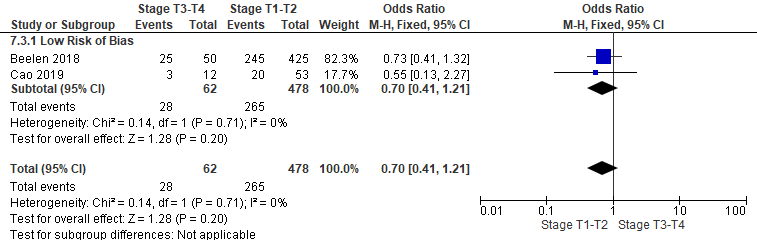
**

**Supplementary Figure 5: Forrest plot of odds ratios for *CCND1* amplification and Stage**

Analysis includes data from all eligible studies regardless of risk of bias (RoB), with segregation into groups based on RoB assessment. Z values indicate the magnitude of association, with p-values <0.05 indicating statistically significant association. Blue squares indicate odds ratio, with values >1 indicative of association of the outcome measure (RFS and OS) with *CCND1* amplification, with strongest association towards the right of the plot. Black lines either side of squares indicate 95% confidence interval (CI). Size of blue boxes is relative to specific study weight with greatest weight given to studies with minimal variance (calculated based on inverse of the variance). Large black diamond represents pooled odds ratio estimate of the above studies. A fixed effects approach was taken. SE – Standard Error. Plots were generated in Review Manager.

**
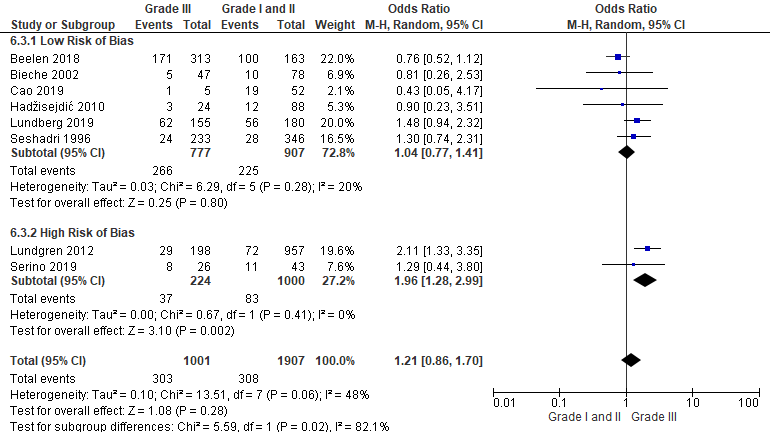
**

**Supplementary Figure 6: Forrest plot of odds ratios for *CCND1* amplification and Grade**

Analysis includes data from all eligible studies regardless of risk of bias (RoB), with segregation into groups based on RoB assessment. Z values indicate the magnitude of association, with p-values <0.05 indicating statistically significant association. Blue squares indicate odds ratio, with values >1 indicative of association of the outcome measure (RFS and OS) with *CCND1* amplification, with strongest association towards the right of the plot. Black lines either side of squares indicate 95% confidence interval (CI). Size of blue boxes is relative to specific study weight with greatest weight given to studies with minimal variance (calculated based on inverse of the variance). Large black diamond represents pooled odds ratio estimate of the above studies. A random effects approach was taken. SE – Standard Error. Plots were generated in Review Manager.
